# Multiomic analysis of lactylation and mitochondria-related genes in hepatocellular carcinoma identified MRPL3 as a new prognostic biomarker

**DOI:** 10.3389/fonc.2024.1511958

**Published:** 2025-01-10

**Authors:** Wenya Xing, Yuanzi Zhou, Qiuzi Long, Nan Yi, Gaoyuan Wang, Rongwei Shi, Jinlong Huang, Xindong Yin, Taiyang Zhu, Shibing Cao

**Affiliations:** ^1^ Department of General Surgery, Affiliated Hospital of Nanjing University of Chinese Medicine, Jiangsu Province Hospital of Chinese Medicine, Nanjing, China; ^2^ Nanjing University of Chinese Medicine, Nanjing, China; ^3^ Department of General Internal Medicine, Affiliated Hospital of Nanjing University of Chinese Medicine, Jiangsu Province Hospital of Chinese Medicine, Nanjing, China

**Keywords:** hepatocellular carcinoma, lactylation, epigenetic regulation, MRPL3, prognostic biomarkers

## Abstract

**Background:**

Recent research has highlighted lactate’s crucial role in epigenetic regulation, particularly by influencing histone modifications that drive the initiation and progression of hepatocellular carcinoma (HCC). While mitochondria are known to regulate tumor behavior, the interaction between lactate metabolism and mitochondrial function in cancer tissues remains underexplored. Understanding this relationship may provide deeper insights into tumor metabolic reprogramming and reveal novel therapeutic targets for HCC and other malignancies.

**Methods:**

We conducted a comprehensive screening of lactylation- and mitochondria-associated genes (LMRGs) in HCC patients, followed by clustering based on these genes. Prognostic outcomes and pathway enrichment were analyzed across the identified clusters. Additionally, we developed a prognostic model based on LMRGs, examining its implications for survival, immune response, and drug sensitivity. *In vitro* experiments were performed to validate the expression patterns and functional role of MRPL3 in HCC.

**Results:**

We developed a prognostic model, named the LMRG model, incorporating three key genes: ACACA, MRPL3, and MRPS23. This model revealed significant differences in survival outcomes, immune responses, and drug sensitivity between patients with high and low LMRG scores. MRPL3 was found to be overexpressed in HCC, playing a critical role in tumor growth and metastasis. These results were further validated through *in vitro* experiments, confirming MRPL3’s role in HCC cell proliferation and invasion.

**Conclusion:**

We created a predictive model, LMRG, and identified MRPL3 as a key biomarker. Our findings suggest that MRPL3 has significant potential as a reliable predictive biomarker for clinical applications in HCC diagnosis and treatment.

## 1 Introduction

As of 2022, primary liver cancer ranks as the sixth most prevalent cancer and the third leading cause of cancer-related deaths globally (1). The predominant subtype of primary liver cancer is hepatocellular carcinoma (HCC), followed by cholangiocarcinoma (CC) (2, 3). The primary risk factors for HCC include chronic infections with hepatitis B virus (HBV) and hepatitis C virus (HCV), as well as alcoholic and non-alcoholic fatty liver diseases (4). Although surgery remains the main treatment for HCC, recurrence and metastasis are common challenges (5). Ablation therapies, such as microwave ablation (MWA), are effective for early-stage HCC (6). Other treatment modalities, including intra-arterial therapy, radiotherapy, and systemic therapies (standard cytotoxic chemotherapy, targeted therapy), offer additional options but are often hindered by drug resistance, hepatic impairment, and tumor resilience mechanisms (7, 8).

The limitations of current therapeutic approaches underscore a critical need to delve deeper into the gene regulatory mechanisms underlying HCC. For instance, resistance to chemotherapy and radiotherapy is frequently linked to dysregulated signaling pathways and epigenetic modifications that enable tumor progression and survival under treatment pressures (9). Moreover, understanding how HCC evolves in the context of its complex etiology and microenvironment could provide novel insights into therapeutic vulnerabilities. Thus, exploring gene regulatory networks is not only vital for uncovering the molecular underpinnings of HCC but also for identifying biomarkers for early diagnosis and developing targeted therapies that circumvent resistance.

In recent years, lactate and mitochondrial function have emerged as critical factors influencing cancer biology, including HCC. While lactate accumulation is a hallmark of altered tumor metabolism under the Warburg effect (10), mitochondria, despite reduced reliance on oxidative phosphorylation in many tumors, remain pivotal in producing reactive oxygen species (ROS) and supporting biosynthetic pathways essential for rapid tumor growth (11). These two elements are intricately linked through metabolic signaling networks, highlighting their potential role in HCC progression and therapeutic resistance.

Lactate, traditionally seen as a glycolysis byproduct, plays a critical role in cancer metabolism. The Warburg effect highlights tumor cells’ reliance on glycolysis, leading to elevated lactate production (10, 12). Beyond metabolism, lactate acts as a regulatory molecule influencing immune modulation (12) and histone lysine lactylation, which translates metabolic signals into transcriptional changes (13, 14). In HCC, Gao et al. showed that K28 lactylation promotes proliferation and metastasis by inhibiting adenylate kinase 2 (AK2) (15), while Xu et al. found that Demethylzeylasteral (DML) suppresses H3K9la and H3K56la lactylation, inhibiting HCC progression (16). Bioinformatics studies by Chen et al. revealed that lactylation-related genes predict HCC prognosis, immunity, mutations, and drug sensitivity (17). These findings position lactylation as a promising epigenetic target in cancer research.

Mitochondria are vital for ATP production via oxidative phosphorylation and play key roles in respiration, metabolism, and apoptosis (18). Their genome encodes components of the electron transport chain (ETC), a major source of reactive oxygen species (ROS), which trigger signaling pathways, promote proliferation, and drive tumor progression (19, 20). The interplay between glycolysis and mitochondrial metabolism regulates tumor microenvironment adaptation, with glycolysis-derived lactate altering mitochondrial functions and mitochondria-generated ROS influencing histone lactylation. Oncogenic factors like c-Myc, HIF-1α, PI3K/Akt, and p53 modulate these interactions, linking mitochondria to HCC progression and therapy resistance (21–24). Prognostic models using mitochondria-related genes, such as those by Zhang B et al. (eight genes) and another study (six genes), highlight mitochondria’s critical role in HCC diagnostics and therapeutics (25, 26).

Pyruvate from glycolysis is converted to lactate under hypoxia, creating an immunosuppressive environment and promoting cancer growth. Lactate also modifies histone lysines, regulating gene expression (27). Leah I. Susser et al. showed that mitochondrial fragmentation increases lactate, driving histone lactylation and M2-like macrophage responses (28), demonstrating a bidirectional link between lactate and mitochondrial dynamics. This highlights a bidirectional relationship where glycolytic intermediates such as lactate influence mitochondrial dynamics, and mitochondrial processes modulate epigenetic reprogramming through lactylation. Thompson’s group revealed lactate activates the electron transport chain in mitochondria, boosting ATP production (29). Jingwei Ma et al. found lithium carbonate enhances T-cell anti-tumor activity by driving lactic acid into mitochondria. However, research on lactylation and mitochondria in HCC remains scarce.

First, we screened for genes associated with lactylation and mitochondria, termed LMRGs and analyzed their differential expression across databases such as The Cancer Genome Atlas (TCGA), Gene Expression Omnibus (GEO), and the International Cancer Genome Consortium (ICGC). Using least absolute shrinkage and selection operator (LASSO) regression, we established an LMRG model to identify prognosis-related genes. We then performed mutational, immunological, and drug sensitivity analyses. Additionally, we selected a key gene, MRPL3, from the model for clinical and immune correlation analyses. By linking lactylation and mitochondrial functions, we aimed to unveil novel regulatory mechanisms and identify actionable biomarkers for HCC, addressing critical gaps in existing research.

## 2 Materials and methods

### 2.1 The source of the LMRGs

Zhao Y et al. first identified histone lactylation and its regulatory role in cellular functions (13). Based on their findings, lactylation-related genes were identified by extracting genes directly reported to be involved in lactylation processes or significantly impacted by lactylation in their study. These genes were included based on their functional relevance to lactylation, as demonstrated through experimental evidence or validated mechanisms. For mitochondria-related genes, we utilized the Human MitoCarta3.0 database, a comprehensive resource cataloging mitochondrial proteins and pathways (30), available at https://www.broadinstitute.org/mitocarta/mitocarta30-inventory-mammalian-mitochondrial-proteins-and-pathways. Genes annotated as mitochondrial components or pathways in MitoCarta3.0 were selected for further analysis. To define the lactylation-mitochondria-related genes (LMRGs), we intersected the identified lactylation-related gene set with the mitochondria-related gene set. This intersection highlighted genes simultaneously associated with lactylation and mitochondrial functions.

### 2.2 Collection of analytical data

We retrieved expression and clinical data about HCC from multiple sources, including TCGA (https://portal.gdc.cancer.gov/), GEO (https://www.ncbi.nlm.nih.gov/geo/), and ICGC (https://dcc.icgc.org/). TCGA’s dataset was notably supplemented with mutation data and copy number variation (CNV) information. The expression and clinical data were subsequently integrated into a matrix file using Strawberry Perl software (version 5.30.0.1). Data for pan-cancer were sourced from the University of California Santa Cruz Xena browser (UCSC Xena) database (http://xena.ucsc.edu/), RNAseq data in TCGA and Genotype-Tissue Expression (GTEx) in TPM format and their corresponding normal tissue data were processed uniformly by the Toil program (31). We have organized the data used and presented it in a tabular form (**Table 1**).

**Table 1:** The clinical features of HCC patients from TCGA, GEO, and ICGC datasets.

Clinical features	Total patients(851)		TCGA(424)		GSE76427(167)		ICGC(260)	
	Number	Percentage(%)	Number	Percentage(%)	Number	Percentage(%)	Number	Percentage(%)
Type								
Tumor	749	88.01%	374	88.21%	115	68.86%	260	100%
Normal	102	11.99%	50	11.79%	52	31.14%	0	0%
Fustat								
Alive	201	26.73%	132	35.01%	23	20.00%	46	17.69%
Dead	551	73.27%	245	64.99%	92	80.00%	214	82.31%
Age								
≤65	398	52.93%	235	62.33%	65	56.52%	98	37.69%
>65	353	46.94%	141	37.40%	50	43.48%	162	62.31%
Unknown	1	0.13%	1	0.27%	0	0%	0	0%
Gender								
Female	212	28.19%	122	32.36%	22	19.13%	68	26.15%
Male	540	71.81%	255	67.64%	93	80.87%	192	73.85%
Stage								
I-II	509	67.69%	262	69.50%	90	78.26%	157	60.38%
III-IV	219	29.12%	91	24.14%	25	21.74%	103	39.62%
Unknown	24	3.19%	24	6.37%	0	0%	0	0%

### 2.3 Recognition of differentially expressed LMRGs

The differentially expressed genes between normal and tumor tissues from TCGA and GEO were identified by the “limma” R package, we set the cutoff criteria for significant fold changes and false discovery rates (FDR) to ensure robust identification of key genes. Specifically, genes with a fold change (|log FC |) > 2 or< 0.5 and an FDR< 0.05 were considered significant. These thresholds were chosen based on widely accepted standards in transcriptomic analysis and were further validated for consistency with the biological relevance of identified genes. Take the intersection of DEGs and LMRGs to get the final differentially expressed LMRGs (DE-LMRGs). For the identified DE-LMRGs, we conducted tumor mutation burden (TMB) analysis by TCGA dataset. The frequency of CNV in DE-LMRGs was calculated based on gene copy number gain and deletion. A functional enrichment analysis of DE-LMRGs was conducted, encompassing Gene Ontology (GO) and Kyoto Encyclopedia of Genes and Genomes (KEGG) analysis, utilizing the “clusterProfiler” R package.

### 2.4 Clustering analysis on the basis of DE-LMRGs

The TCGA and GEO expression and survival data were merged and batch effects were removed using the “ComBat function” of the “SVA” R package. Based on the DE-LMRGs, we conducted a clustering analysis of the merged data by the “ConsensusClusterPlus” R package, the HCC patients were thus classified into different LMRG clusters. To verify the accuracy of the clustering, Principal Component Analysis (PCA) was performed to show the overall differences between different clusters. We analyzed the survival differences between patients in different clusters and plotted the survival curves using the “survival” R package. By the “pheatmap” R package, a heatmap of gene expression linked to clinical information was created. Gene Set Variation Analysis (GSVA) based on the “c2.cp.kegg.symbols.gmt” gene set was performed to explore the functional pathways of HCC between different clusters. To determine the immune cell content between different clusters, Single Sample Gene Set Enrichment Analysis (ssGSEA) was subsequently conducted. Both processes are realized by the “GSVA” and “GSEABase” R packages.

### 2.5 Construction and validation of the LMRG prognostic model

In order to further explore the role of LMRGs on prognosis, we developed the LMRG score. Regarding the clinical features of HCC, univariate Cox (uniCox) analysis was performed on the merged data. Then, we added the ICGC data as an external test set and the merged data as an internal training set to build the LMRG model using LASSO regression, subsequently the LMRG score is calculated according to the following formula:


$$LMRG\;\;Score=\sum \;coef\;\left(LMRGs\right)\;\times \;exp\;\left(LMRGs\right)$$

The median LMRG score was used to stratify HCC patients into high- and low-LMRG-score groups. Subsequently, risk plots and expression heatmaps were constructed, leveraging the prognosis-related LMRGs, to facilitate a more intuitive understanding of the disparities in gene expression profiles between the distinct LMRG groups. We did Kaplan-Meier (KM) analysis using the “survminer” and “survival” R packages.

The Receiver Operating Characteristic (ROC) curve was employed to demonstrate the predictive capacity of the model, and Principal Component Analysis (PCA) and t-distribution Stochastic Neighbor Embedding (t-SNE) were designed to better distinguish different LMRG groups, which were implemented by the “timeROC” and “Rtsne” R packages, respectively.

### 2.6 Prognosis analysis of the clinical

To ascertain the correlation between the LMRG score and clinical status, we performed both uniCox and multivariate Cox (muiCox) analyses. Subsequently, the LMRG score of HCC patients and their clinical features were finely mapped to 1-, 3-, and 5-year overall survival with the “rms” R package. To validate the precision and reliability of the nomogram for clinical utilization, in-depth calibration curves were generated and analyzed. Furthermore, the discrepancies in LMRG score were examined in relation to various clinical characteristics, which were represented in box-and-line plots.

### 2.7 Correlation between the LMRG model and LMRG clusters

We investigated the differences in LMRG score between the two clusters to define whether the LMRG model could be applied to clustering. Then, correlations between the LMRG clusters, LMRG score, and survival outcome were assessed and depicted by using the “galluvial” R package.

### 2.8 PPI network and enrichment analysis

A Protein-Protein Interaction (PPI) network of prognosis-related LMRGs and DE-LMRGs was constructed through the STRING website (https://cn.string-db.org/cgi/input.pl). Furthermore, we performed GO and KEGG enrichment analysis on the prognosis-related LMRGs to elucidate the underlying pathways that are pertinent to our model.

### 2.9 Immunological and tumor stem cell analysis

Immune cell infiltration levels across all samples were quantitatively assessed utilizing the CIBERSORT algorithm, permitting us to subsequently evaluate the relationship between the LMRG score and derived immune score. The correlations between prognosis-related LMRGs, LMRG score, and immune cells were also analyzed. These steps were visualized through the “ggpubr,” “ggplot,” and “reshape2” R packages. We scored the tumor microenvironment (TME) by the ESTIMATE algorithm on three dimensions: StromalScore, ImmuneScore, and ESTIMATEScore. And differences in the distributions of high- and low-LMRG-score groups were visually represented and compared using violin plots. In addition, we conducted tumor stem cell correlation analysis of the LMRG model based on RNA stemness scores (RNAss).

### 2.10 Chemotherapy drug sensitivity analysis

Data on drugs were taken from the Genomics of Drug Sensitivity in Cancer (GDSC) website (https://www.cancerrxgene.org). The half-maximal inhibitory concentration (IC50) was employed to evaluate drug sensitivity between the high- and low-LMRG-score groups using the “oncoPredict” R package.

### 2.11 Expression and prognostic analysis of MRPL3

We extracted the ENSG00000114686.8 (MRPL3) molecule from the UCSC database, performed log2^(TPM+1)^ transformation of the expression values, and analyzed the data differences using the “stats” R package. Moreover, we extracted this molecule in the TCGA-ALL database according to the same method and performed a paired-sample difference analysis using the same R package. We obtained the TCGA prognostic dataset (32), excluding samples with a follow-up duration of less than 30 days, and employed the “survival” package to construct a Cox proportional hazards regression model, which aimed to elucidate the correlation between gene expression profiles and prognosis within each tumor type. The outcomes of this analysis were then graphically represented using the “ggplot2” R package.

### 2.12 Single-cell sequencing analysis of MRPL3

This step was achieved on the Tumor Immunity Single Cell Center 2 (TISCH2) flat (http://tisch.compgenomics.org/home/). The GEO scRNA-seq dataset (GSE140228), which contains 62,530 cells from 5 HCC tissues, was selected for this study, and the scRNA-seq was performed using a 10x Genomics platform. The “NormalizeData” function in “Seurat” was used to normalize the data. The raw count (UMI) in each cell was 10,000.

### 2.13 Cell culture and tissue collection of HCC

The human hepatic normal cell line (THLE2) and five HCC cell lines (Huh7, Hep3B, HepG2, HCC-LM3, Li-7) for this experiment were cultured using DMEM medium (Gibco, USA) containing 10% fetal bovine serum (BiologicalIndustries, Israel). In addition, 30 pairs of primary HCC tumors and adjacent tissues were collected at Jiangsu Province Hospital of Chinese Medicine (Nanjing, China). The research project was granted ethical approval by the Ethics Committee of Jiangsu Province Hospital of Chinese Medicine (No. 2023NL-132-01), and written informed consent was obtained from all participants.

### 2.14 Real-time reverse transcriptase PCR

RNA was extracted from tissues and cells using TRIzol reagent (Gibco, USA) and Complementary DNA (cDNA) synthesis was performed using PrimeScript^®^RT Kit (TaKaRa, Japan). Subsequently, a fluorescent quantitative PCR instrument and primers were used for RT-qPCR analysis of this cDNA, which was repeated thrice per sample.

### 2.15 Knockdown of MRPL3 via transfection

A specific shRNA, designed to downregulate MRPL3 expression, was synthesized by GenePharma Co., Ltd. (Shanghai, China), with pLKO.1 serving as a control. Lentiviral packaging and transfection were then performed in 293T cells. The concentrated lentivirus, along with hexadimethrine bromide (Beyotime, China), was introduced into HCC cell lines (Hep3B, HCC-LM3). The transfected stable cell lines were selected using puromycin (Solarbio, China). Cell Counting Kit-8 assay (CCK-8).

The cells were categorized into sh-NC, sh-MRPL3 of the Hep3B, and HCC-LM3 after transfection and were uniformly planted in 96-well plates, respectively. After 24h of incubation, 100μl of fresh DMEM medium and 10μl of CCK8 solution were subsequently added to every well. The plates were incubated again for 4h, and then the absorbance at 450nm was measured on an enzyme-linked immunoassay detector (Tecan, Switzerland).

### 2.16 Flow cytometric apoptosis assay

Hep3B, HCC-LM3 cells were incubated with RNase A and propidium iodide (Sigma, USA) for 15 min at 20°C away from light. Then, the cells were treated with Annexin V-FITC/PI Apoptosis Detection Kit (Roche, Switzerland) in accordance with the instructions provided by the manufacturer. The distribution of cell cycle phases was ultimately analyzed using flow cytometry (BD, USA), and the apoptosis level was analyzed.

### 2.17 Western blot assay

Total protein extraction from Hep3B cells and 5 pairs of patients’ tissues was conducted using a protein extraction kit (Beyotime, China), and determination of protein concentration was performed by a BCA kit (Beyotime). The extracted proteins were separated on a 10% SDS-PAGE gel and transferred to PVDF membranes (Millipore, USA). The membranes with cell proteins were blocked and incubated with primary antibodies (Cleaved-Caspase3, Cleaved-Caspase9, Bcl-2, E-cadherin, vimentin, GAPDH) overnight, while the membranes with tissue proteins were blocked and incubated with MRPL3 antibody overnight. After incubation with secondary antibodies (Goat anti-rabbit IgG (h+l), HRP), bands were detected by chemiluminescence using an imaging system. All antibodies used were purchased from Affinity Biosciences (USA).

### 2.18 Wound-healing assay

After spreading Hep3B, HCC-LM3 cells to complete fusion, a line of cells was removed by scratching at the bottom of the culture dish using a sterile gun tip. The influence of cell proliferation was excluded by changing the serum-free medium, and the healing of the scratch was photographed and recorded at predetermined time points (0h, 48h). The capacity of the cells to migrate was evaluated by quantifying the alteration in the width of the scratch.

### 2.19 Cell migration and invasion assays

Matrigel (BD, USA) was applied to the upper layer of Transwell chambers (Costar, USA) to test the invasive ability of cells; the step is not necessary when detecting cell migration ability. The experimental cell lines were inoculated into the upper layer of Transwell chambers containing serum-free medium, and the lower layer was placed in a 10% FBS medium for chemotaxis. Following a 24-hour incubation period, non-migrating or non-invasive cells were meticulously removed with cotton swabs. Subsequently, methanol was employed to immobilize the remaining cells, which were then stained with crystal violet. Three views of each chamber were selected and counted under a microscope (Olympus, Japan) to quantify the capacity for cell migration and invasion.

### 2.20 Statistical analysis

The statistical analyses in this study were conducted using R software (version 4.2.1). The specific statistical tests employed include Student’s t-test for comparing two groups, one-way ANOVA followed by Tukey’s *post hoc* test for multiple group comparisons, and Kaplan-Meier survival analysis with the log-rank test for survival comparisons. The rationale for selecting these tests was to ensure that the methods align with the data distribution and study objectives. For instance, the t-test and ANOVA were chosen based on the assumption of normal distribution, which was verified using the Shapiro-Wilk test prior to analysis. For non-normally distributed data, non-parametric tests such as the Mann-Whitney U test or Kruskal-Wallis test were applied as appropriate.

Additionally, Pearson’s or Spearman’s correlation analyses were conducted depending on the data distribution to explore relationships between variables. To mitigate the risk of type I errors in multiple comparisons, we applied the Benjamini-Hochberg procedure to adjust p-values when necessary. The statistical significance threshold was set at p<0.05 for all tests. Data are presented as mean ± standard deviation (SD) unless otherwise specified. Visualizations, including scatter plots, boxplots, and Kaplan-Meier survival curves, were generated using GraphPad Prism version 9 (GraphPad Software, San Diego, CA, USA) to enhance the clarity and reproducibility of the results.

## 3 Results

### 3.1 Identification and functional analysis of lactylation-mitochondria-related genes in HCC

**Figure 1:**
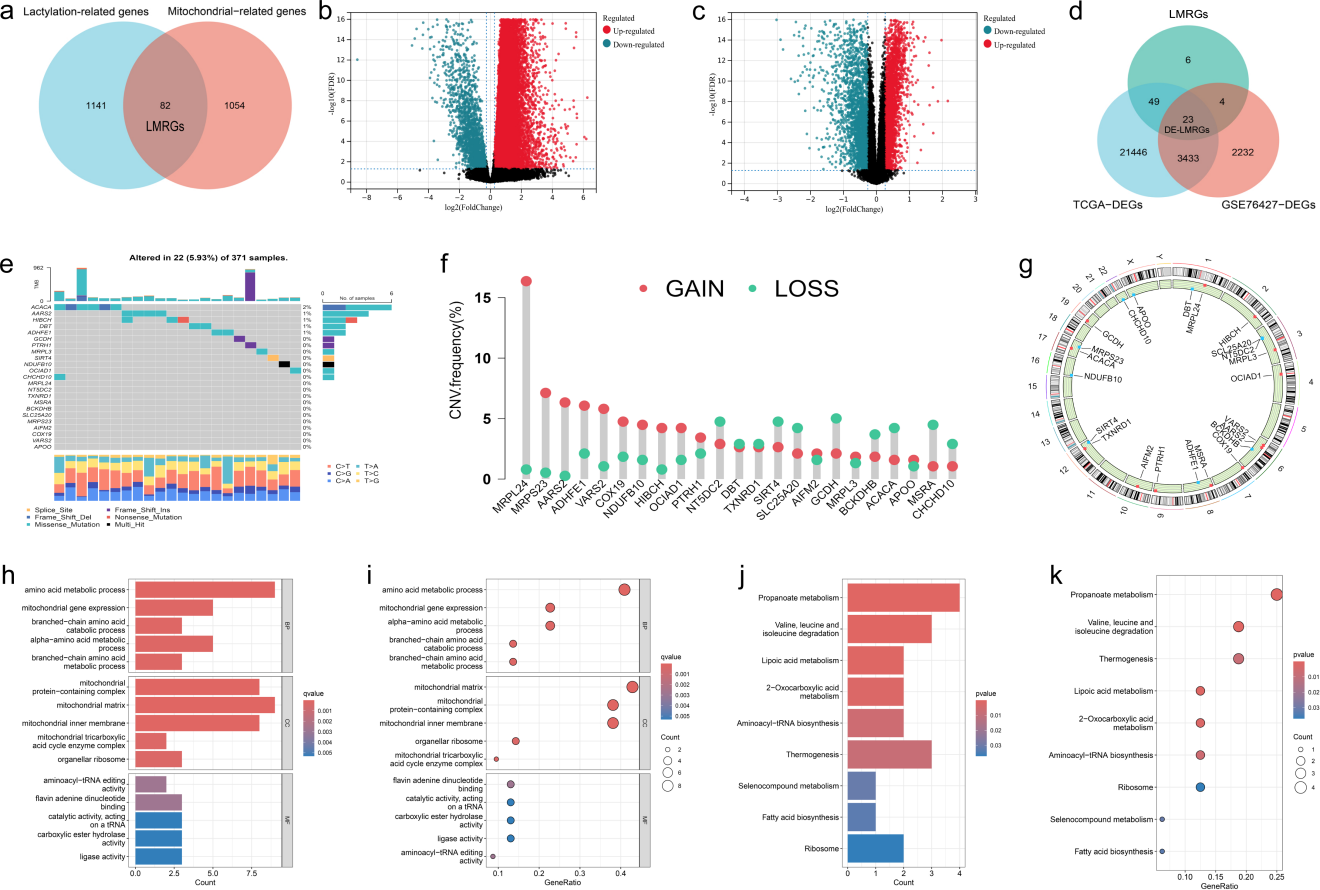
Identification and Functional Enrichment Analysis of Lactylation-Mitochondria-Related Genes (LMRGs) in HCC. **(A)** Venn plot of LMRGs. **(B)** Volcano plot of gene expression variation between hepatic normal and HCC tissues in TCGA dataset and **(C)** in GEO dataset. **(D)** Identification of DE-LMRGs. **(E)** Waterfall plot of mutation frequencies and types of DE-LMRGs in HCC. **(F)** Frequency of copy number variation in DE-LMRGs. **(G)** Circos plots of chromosome distributions among DE-LMRGs. **(H, I)** GO enrichment analysis of DE-LMRGs. **(J, K)** KEGG pathway enrichment analysis of DE-LMRGs.

We searched for 1223 lactylation-related genes from the Supplementary file of Zhao’s article (33) and 1136 mitochondria-related genes from the MitoCarta website, taking the intersection of the two, and ended up with 82 LMRGs (**Figure 1A**). The 82 genes identified in this study are presented in detail in **Supplementary Table S1**. In addition, gene expressions between normal and tumor samples in TCGA and GEO datasets were analyzed separately, resulting in 24,951 differential genes in TCGA and 5,692 differential genes in GSE76427 (**Figures 1B, C**). Taking the overlap of these differentially expressed genes with LMRG, we finally gained 23 DE-LMRGs (**Figure 1D**).

To initially explore LMRGs, we first analyzed the TMB and CNV incidence of DE-LMRGs in samples of HCC (**Figures 1E, F**). As shown in the figure, the mutation frequency of these genes was low and there was no obvious consistency in copy number variation, as we visualized the variation of DE-LMRGs on specific chromosomes (**Figure 1G**). Next, we analyzed the enrichment, and DE-LMRGs were predominantly enriched within amino acid metabolic process under biological processes (BP); mitochondrial matrix under cellular components (CC), and flavin adenine dinucleotide binging under molecule function (MF) (**Figures 1H, I**). The predominant enrichment of KEGG pathways was observed in propanoate metabolism and lipoic acid metabolism (**Figures 1J, K**).

### 3.2 Clustering analysis and immune profiling of LMRG clusters in HCC

**Figure 2:**
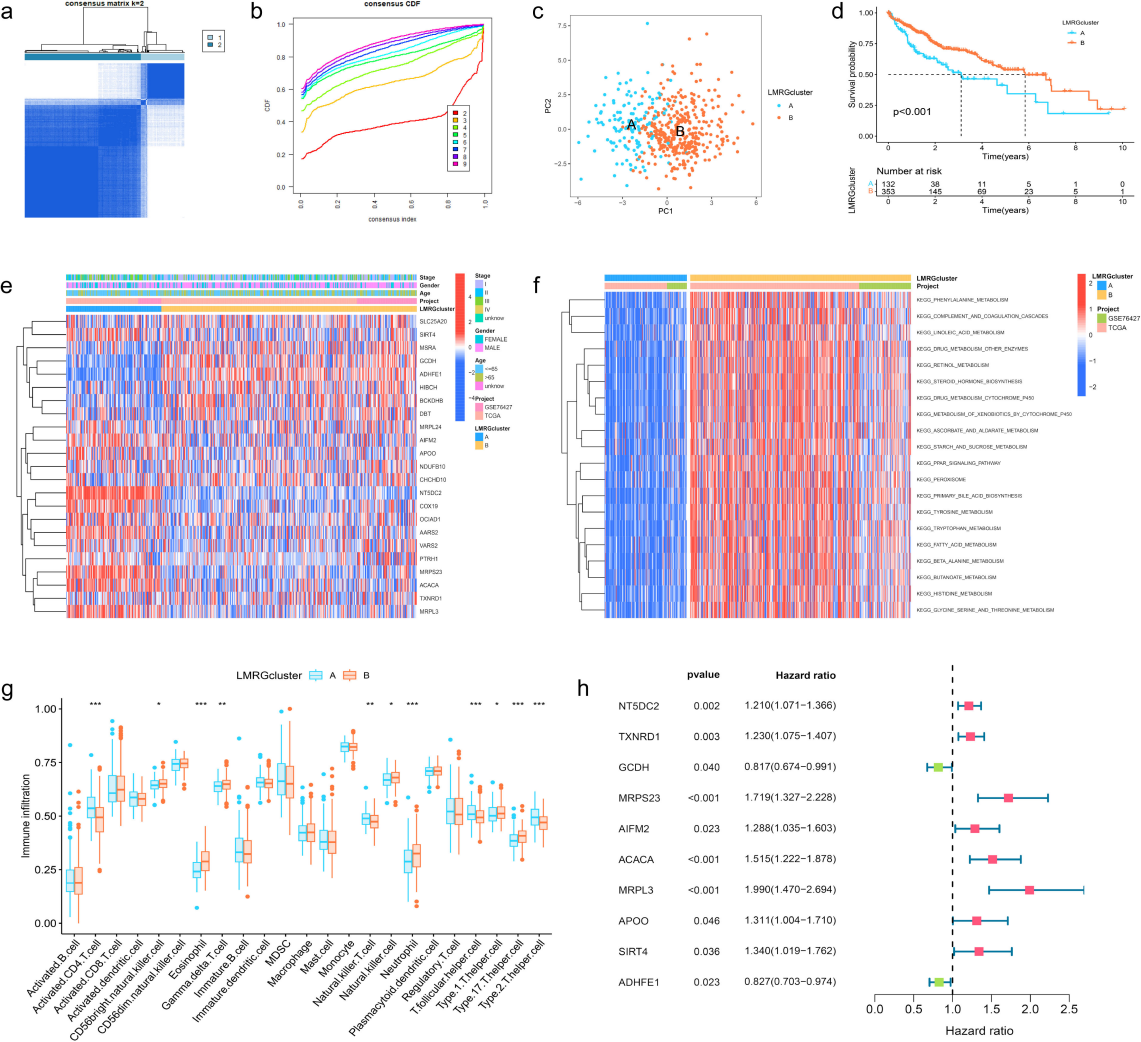
Clustering, Immune Profiling, and Prognostic Analysis of LMRG Clusters in HCC. **(A)** The patients were divided into 2 clusters. **(B)** CDF curves when k=2-9. **(C)** PCA plot showing independence between two clusters. **(D)** Survival analysis between LMRG clusters. **(E)** Heatmap of DE-LMRGs expression, clusters, and clinical features. **(F)** GSVA analysis of the KEGG pathways in LMRG clusters. **(G)** Immune infiltration level of LMRG clusters (*p<0.05; **p<0.01; ***p<0.001). **(H)** Forest plot of univariate Cox regression analysis for DE-LMRGs.

The HCC samples from TCGA and GSE76427 datasets were merged and batch-corrected. After that, we clustered the merged data, and the difference between LMRG clusters was clearest when the number of groups was 2 (**Figures 2A, B**), and PCA results showed that the samples between the two clusters could be discerned with clarity (**Figure 2C**). In terms of OS, there were significant differences between LMRG clusters (p<0.01) (**Figure 2D**). Finally, we plotted the heatmap of gene expression and clinical features (**Figure 2E**).

GSVA analysis was performed between two clusters and demonstrated that most of the differential pathways between the clusters were related to acid metabolism (**Figure 2F**). In addition, we quantified the relative amounts of 23 different immune cell types in two LMRG clusters by ssGSEA analysis. Results showed that most of the differences between the clusters were in T cell types (**Figure 2G**).

### 3.3 Prognostic analysis and risk scoring model based on LMRGs in HCC

To identify LMRGs associated with prognosis, we performed uniCox analysis on the merged data. As results revealed, of 23 DE-LMRGs, 10 genes were associated with HCC prognosis, and 8 of them (NT5DC2, TXNRD1, MRPS23, AIFM2, ACACA, MRPL3, APOO, SIRT4) were associated with poor prognosis, and 2 of them (GCDH, ADHFE1) were associated with good prognosis (**Figure 2H**).

**Figure 3:**
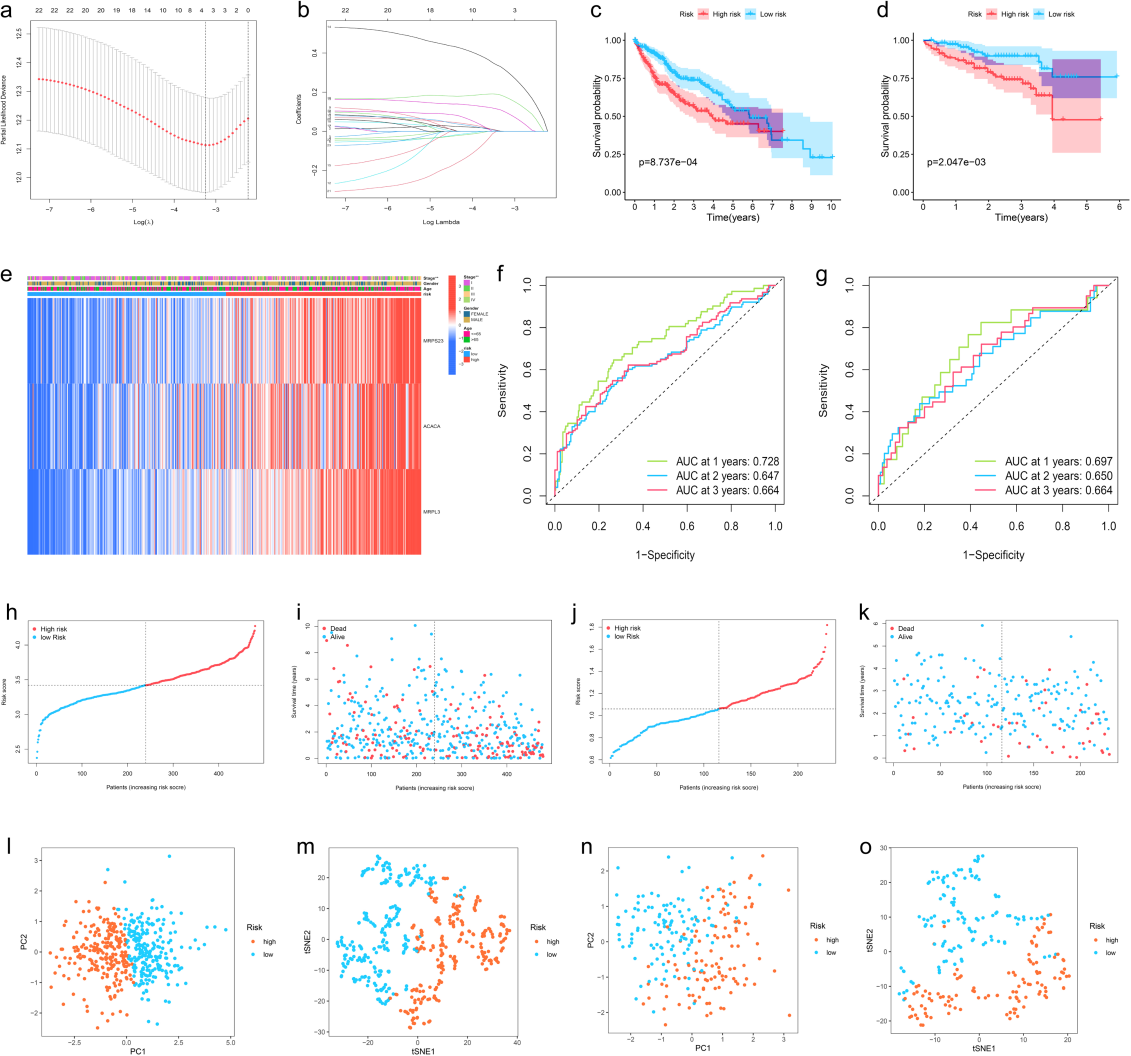
Development, Validation, and Visualization of the LMRG-Based Prognostic Risk Model in HCC. **(A)** Selection of the optimal penalty parameter for LASSO regression. **(B)** LASSO coefficient configuration. **(C)** Kaplan-Meier survival curves between high- and low-LMRG-score groups in the internal training set and **(D)** in the ICGC external test set. **(E)** Heatmap of prognosis-related LMRGs expression in the training set. **(F)** ROC curves for OS in the training set and **(G)** in the ICGC test set. **(H, I)** Patients’ LMRG score distribution, survival status, and time distribution in the training set and **(J, K)** in the ICGC test set. **(L, M)** PCA and t-NSE plot in the training set and **(N, O)** in the ICGC test set.

To avoid LMRG model overfitting, LASSO regression was applied for further screening. The 10 genes mentioned above associated with prognosis were assigned coefficients, and 3 prognosis-related LMRGs (ACACA, MRPL3, MRPS23) were screened by lambda.min (**Figures 3A, B**). Subsequently, the LMRG score for each sample is calculated utilizing the following methodology: LMRG Score = [exp (ACACA)*0.0798] +[exp(MRPL3)*0.2829]+[exp(MRPS23)*0.1586]. The sample was categorized into the high- and low-LMRG-score groups, whereby the median score was used as the cut-off point. Expression of these 3 prognosis-related LMRGs in different LMRG groups is demonstrated by the heatmap (**Figure 3E**).

### 3.4 Validation of the LMRG model validation and clinical application of the LMRG-based prognostic model in HCC

The above-merged data was taken as the internal training set, and another HCC data from the ICGC dataset was used as the external test set; the samples in the test set were also classified into high- and low-LMRG-score groups in accordance with the above methodology. The KM curves demonstrated that the high-LMRG-score group exhibited a poorer prognosis than the low-LMRG-score group in both the training and test sets (**Figures 3C, D**). Furthermore, the risk curves and survival status plots illustrated the risk and survival status of the samples in the training and test sets (**Figures 3H-K**). Next, we validated the model using ROC curves, PCA analysis, and t-SNE analysis, respectively. We visualized the Area Under Curve (AUC) values for survival times of 1-, 2-, and 3-year, which were 0.728, 0.647, and 0.664 for the training set and 0.697, 0.650, and 0.664 for the test set (**Figures 3F, G**). PCA and t-SNE plots showed little overlap between high- and low-LMRG-score groups and a significant tendency toward clustering within the two groups (**Figures 3L-O**).

**Figure 4:**
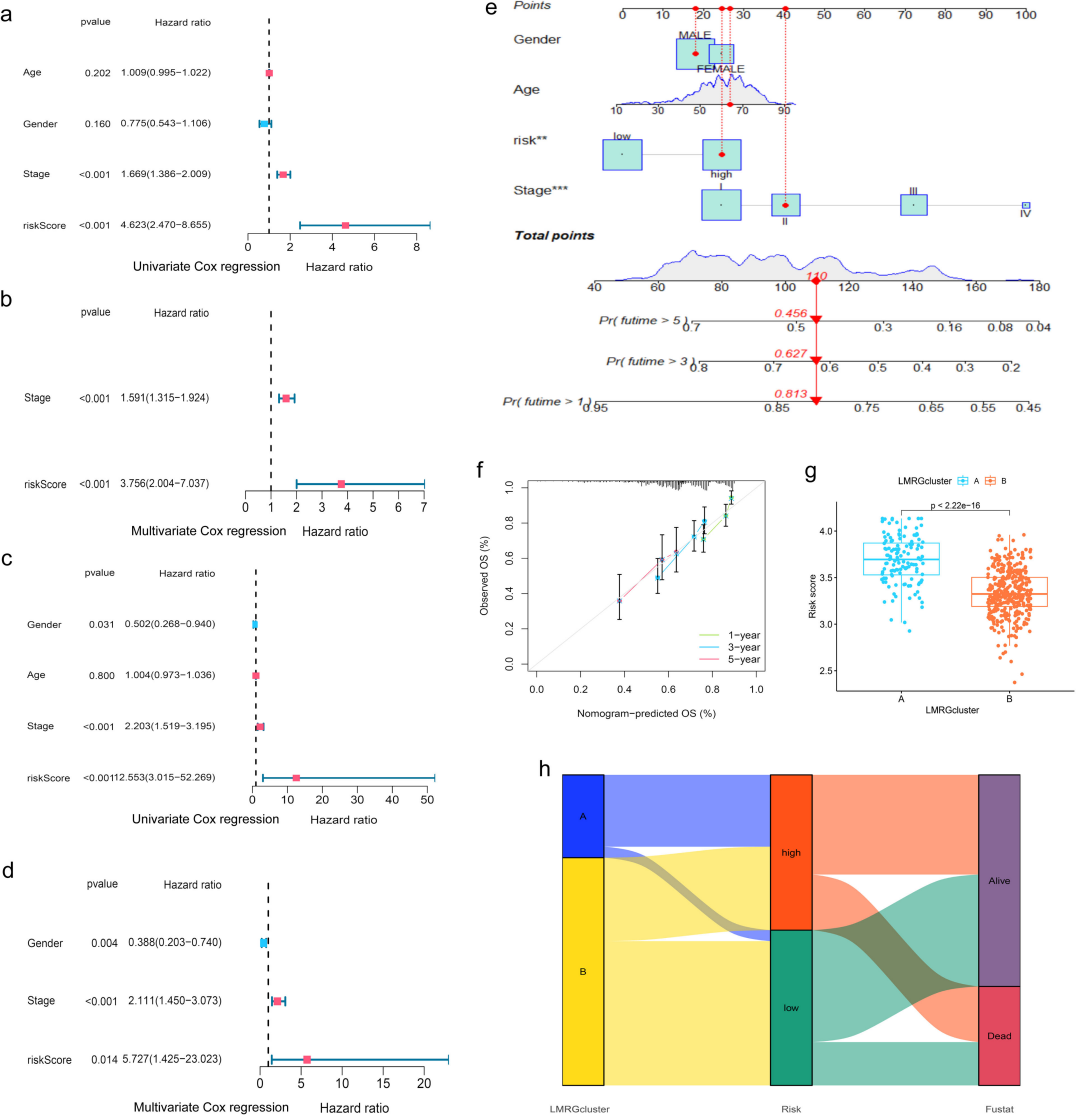
Evaluation of the prognostic efficiency of the LMRG model. **(A, B)** Cox analysis in the training group. **(C, D)** Cox analysis in the test group. **(E)** Nomogram Predicts Patient Survival at 1, 3, and 5 years. **(F)** Calibration of Nomogram. **(G)** Distribution profile of LMRG score in two LMRG clusters. **(H)** Sankey plot of LMRG clusters, LMRG score, and survival outcome. **p<0.01; ***p<0.001.

Moreover, when the clinical features of HCC were analyzed by uniCox and multiCox in the training set, we discovered that “stage” and “LMRG Score” were independently associated with a poor prognosis for patients with HCC (**Figures 4A, B**), and this result was verified in the test set (**Figures 4C, D**).

When comparing the differences in LMRG score across various clinical features, we found that age and gender factors did not have a statistically significant impact on LMRG score. However, tumor stage significantly influenced the LMRG score, demonstrating notable statistical differences (**Supplementary Figure S1**).

Finally, the nomogram based on the LMRG model could predict the 1-, 3-, and 5-year survival rates of HCC patients (**Figure 4E**). Moreover, the calibration curves demonstrated the accuracy and reliability of the aforementioned predictions (**Figure 4F**).

Additionally, we investigated the correlation between the LMRG clusters and the LMRG model, observing a statistically significant difference in LMRG score between the two clusters (p<2.22e-16) (**Figure 4G**). LMRG clusters, LMRG score, and survival status of HCC patients can be visualized by the Sankey plot (**Figure 4H**).

### 3.5 Immune profiling, tumor microenvironment, and functional enrichment of the LMRG model in HCC

**Figure 5:**
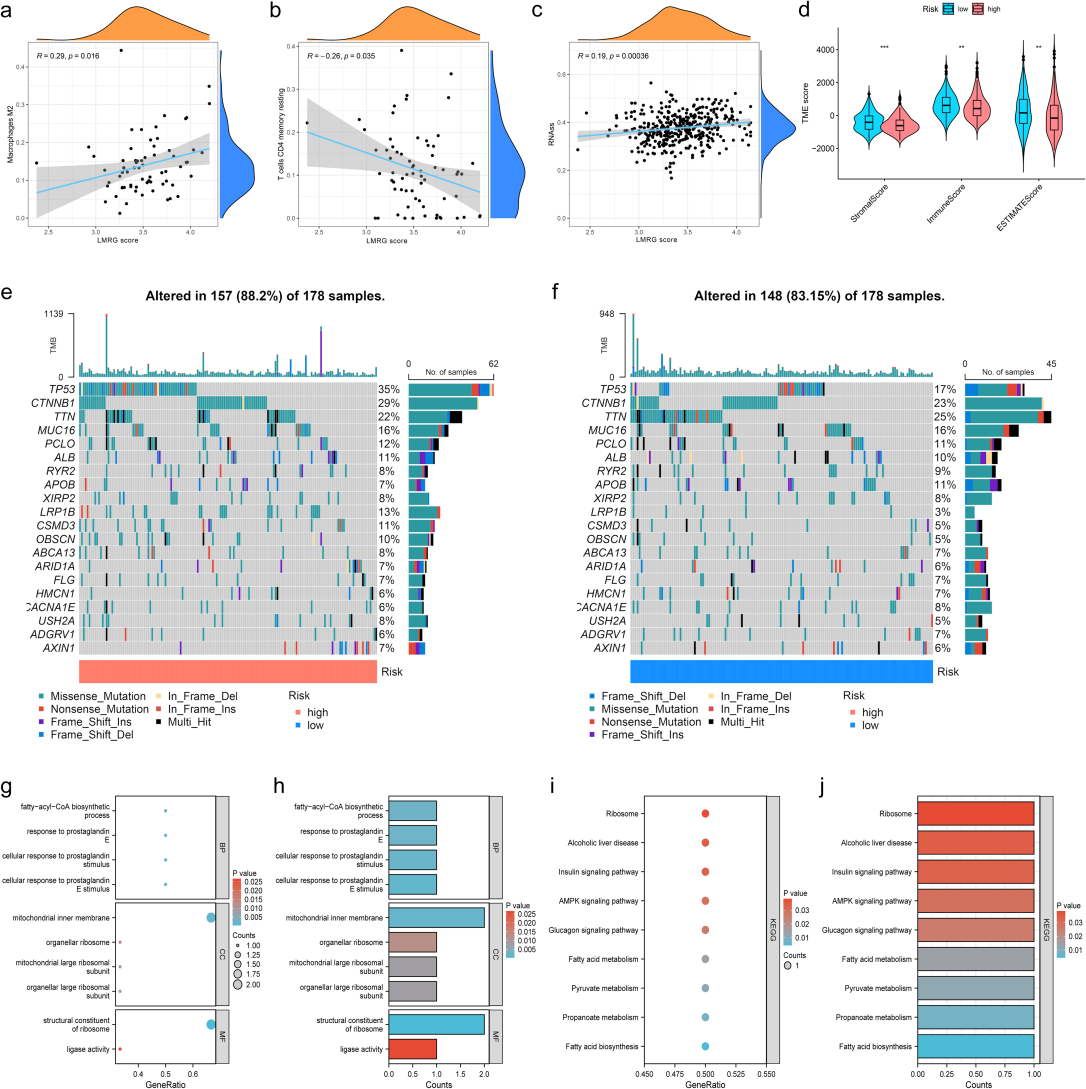
Immunization, mutation and enrichment analysis of the LMRG model. **(A)** M2 immune cell correlation. **(B)** CD4 T-cell immune cell correlation. **(C)** Correlation between the LMRG score and RNAss. **(D)** TME scores between the high- and low-LMRG-score groups. **(E, F)** Waterfall plot of mutation frequencies for the LMRG model. **(G, H)** GO enrichment analysis of prognosis-related LMRGs. **(I, J)** KEGG pathway enrichment analysis of prognosis-related LMRGs. **p<0.01; ***p<0.001.

In order to investigate the relationship between the LMRG model and tumor immune cells, we performed sample immune cell score assignments using the CIBERSOFT algorithm and found that the LMRG score showed a positive correlation with Macrophages M2 cell but a negative correlation with T cells CD4 memory resting (**Figures 5A, B**). However, no significant correlation was found in the remaining cells. The overall correlation plot is shown in the **Supplementary Material** (**Supplementary Figure S2**). Next, the TME analysis revealed that the low-LMRG-score group exhibited elevated StromalScore, ImmuneScore, and ESTIMATEScore values (**Figure 5D**). Stem cell analysis reveals a positive correlation between RNAss and LMRG score (**Figure 5C**). The gene mutation data of HCC from TCGA were visualized between different LMRG groups by waterfall plots, with TP53, CTNNB1, TNN, MUC16, and PCLO being the most commonly mutated genes (**Figures 5E, F**). Although 157 of the 178 (88.2%) samples in the high-LMRG-score group had tumor mutations, compared to 148 (83.15%) in the low-LMRG-score samples, the results revealed no statistically significant differences between the two groups (**Supplementary Figure S3**). The 3 prognosis-related LMRGs in the model were analyzed for enrichment and were mainly enriched for the fatty-acyl-CoA biosynthetic process in BP, for the mitochondrial inner membrane in CC, and for the structural constituent of the ribosome in MF (**Figures 5G, H**). In terms of pathway enrichment, they were mainly present in AMPK signaling, Pyruvate metabolism, and Propanoate metabolism pathways (**Figures 5I, J**).

### 3.6 Identification of drug sensitivities associated with the LMRG model in HCC

**Figure 6:**
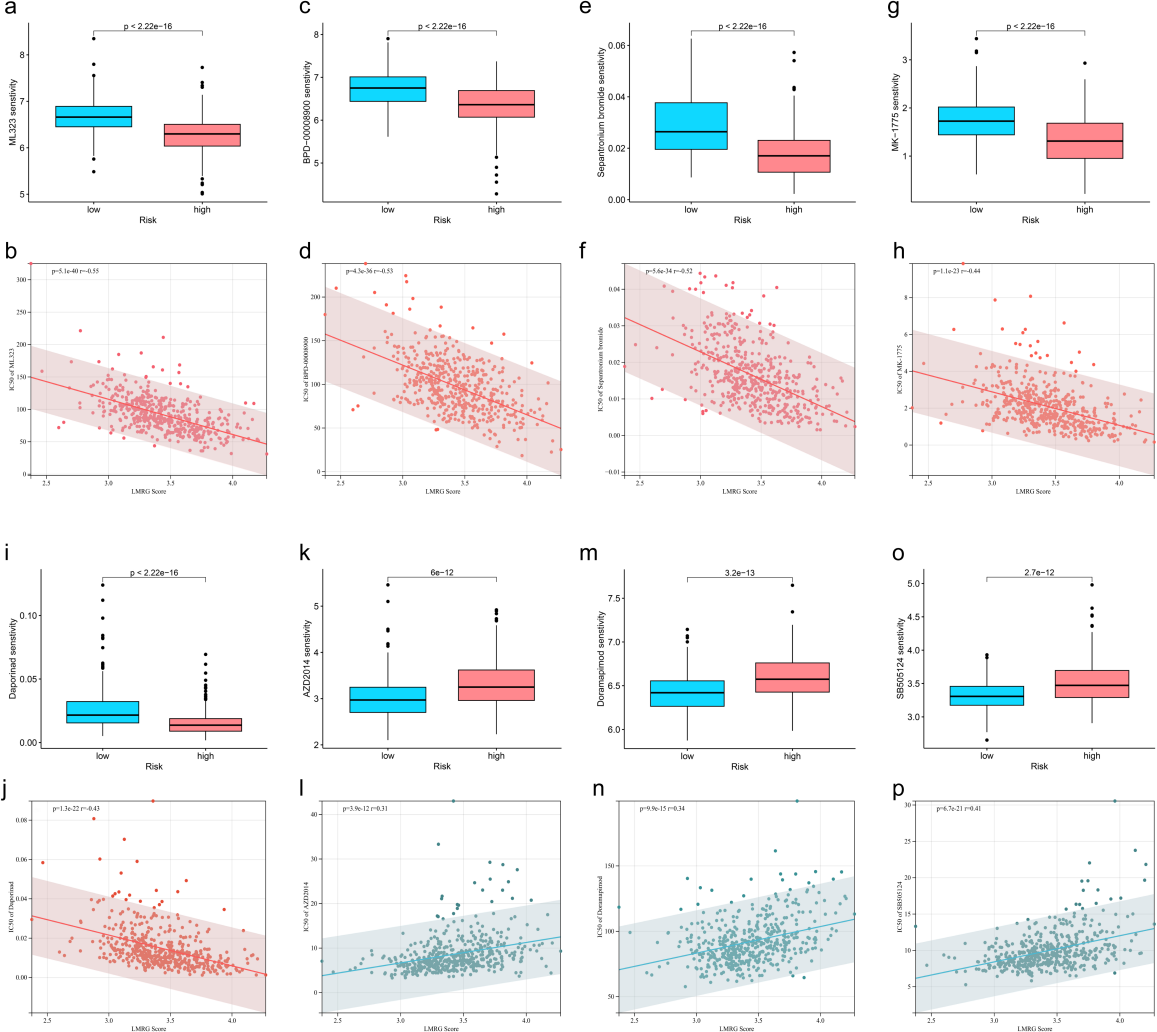
Association between LMRG score and susceptibility to chemotherapy. **(A, B)** ML323, **(C, D)** BPD-00008900, **(E, F)** Sepantronium bromide, **(G, H)** MK-1775, **(I, J)** Selumetinib, **(K, L)** AZD2014, **(M, N)** Doramapimod, **(O, P)** SB505124.

To find effective drugs for the treatment of HCC, we calculated IC50 values of 198 chemotherapeutic drugs in HCC samples and identified 86 drugs with significant differences. We listed 8 drugs, 5 (ML323, BPD-00008900, Sepantronium bromide, MK-1775, Daporinad) of which had elevated IC50 values in the low-LMRG-score group, thus more sensitive to the treatment of high-LMRG-score patients (**Figures 6A-J**). And 3 (AZD2014, Doramapimod, SB505124) of which were more favorable for the treatment of patients in the low-LMRG-score group (**Figures 6K-P**). Other drugs sensitive to HCC are detailed in the **Supplementary Material** (**Supplementary Table S2**).

### 3.7 Visualization of immune cell distribution and MRPL3 expression in HCC samples

**Figure 7:**
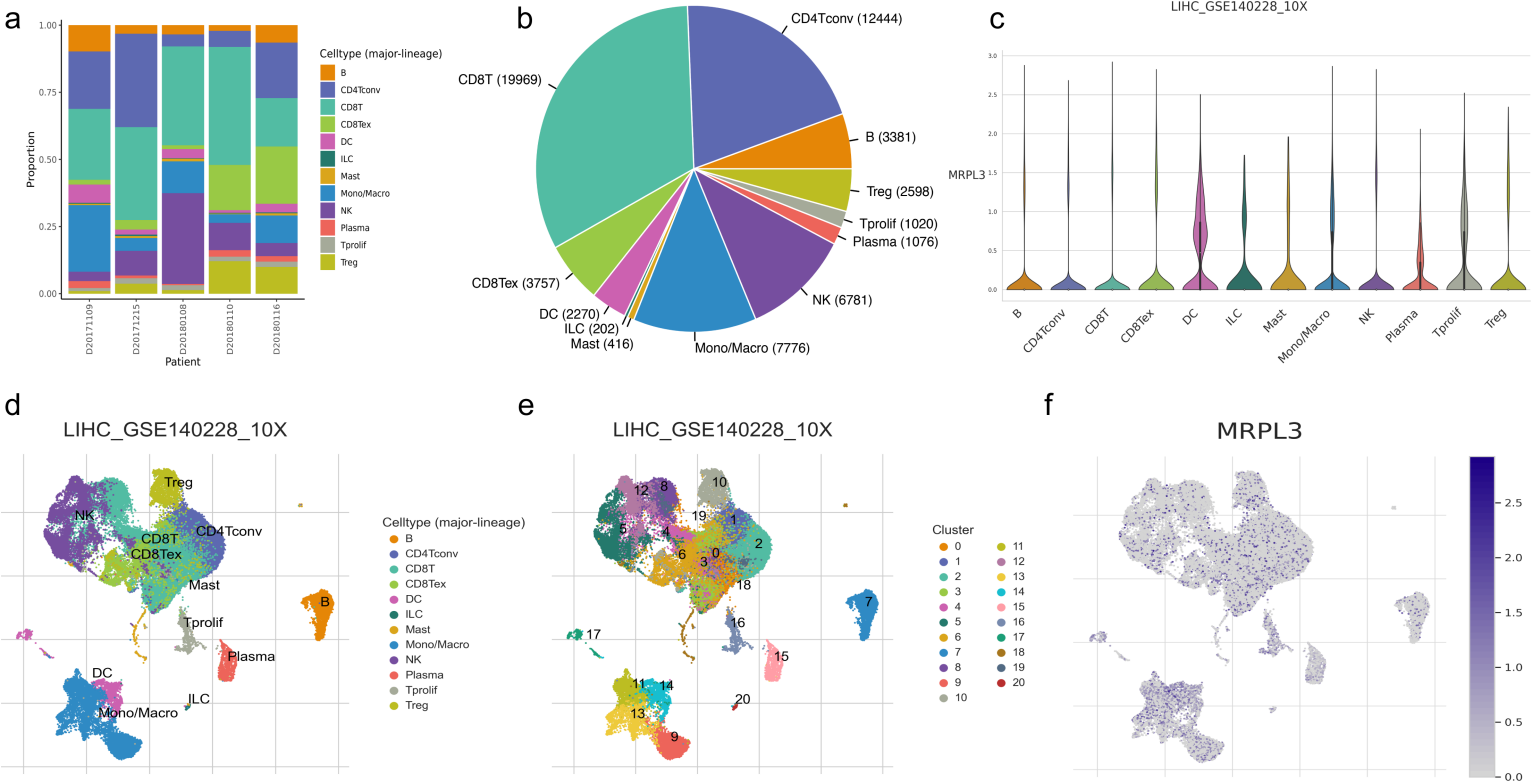
Immune cell composition and mrpl3 expression analysis in HCC by single-cell sequencing analysis. **(A, B)** Content of various cell types. **(C)** Expression of MRPL3 in various immune cells. **(D, E)** Two-dimensional distribution of cells in tissues. **(F)** Two-dimensional distribution of MRPL3 in tissues.

We selected GSE140228 for visualization, a chip containing 5 samples, and first showed the percentage of each cell in each as well as in the total sample, found that CD8T and CD4T cells have high occupancy content (**Figures 7A, B**). By descending to two dimensions, the distribution of immune cells can be observed (**Figures 7D, E**). The distribution of MRPL3 was then demonstrated as well, and it was found to be predominantly aggregated in DC, ILC, Plasma, and Tprolif cells (**Figures 7C, F**).

### 3.8 MRPL3 as a prognostic biomarker and therapeutic target in HCC: expression patterns, survival analysis, and experimental validation

**Figure 8:**
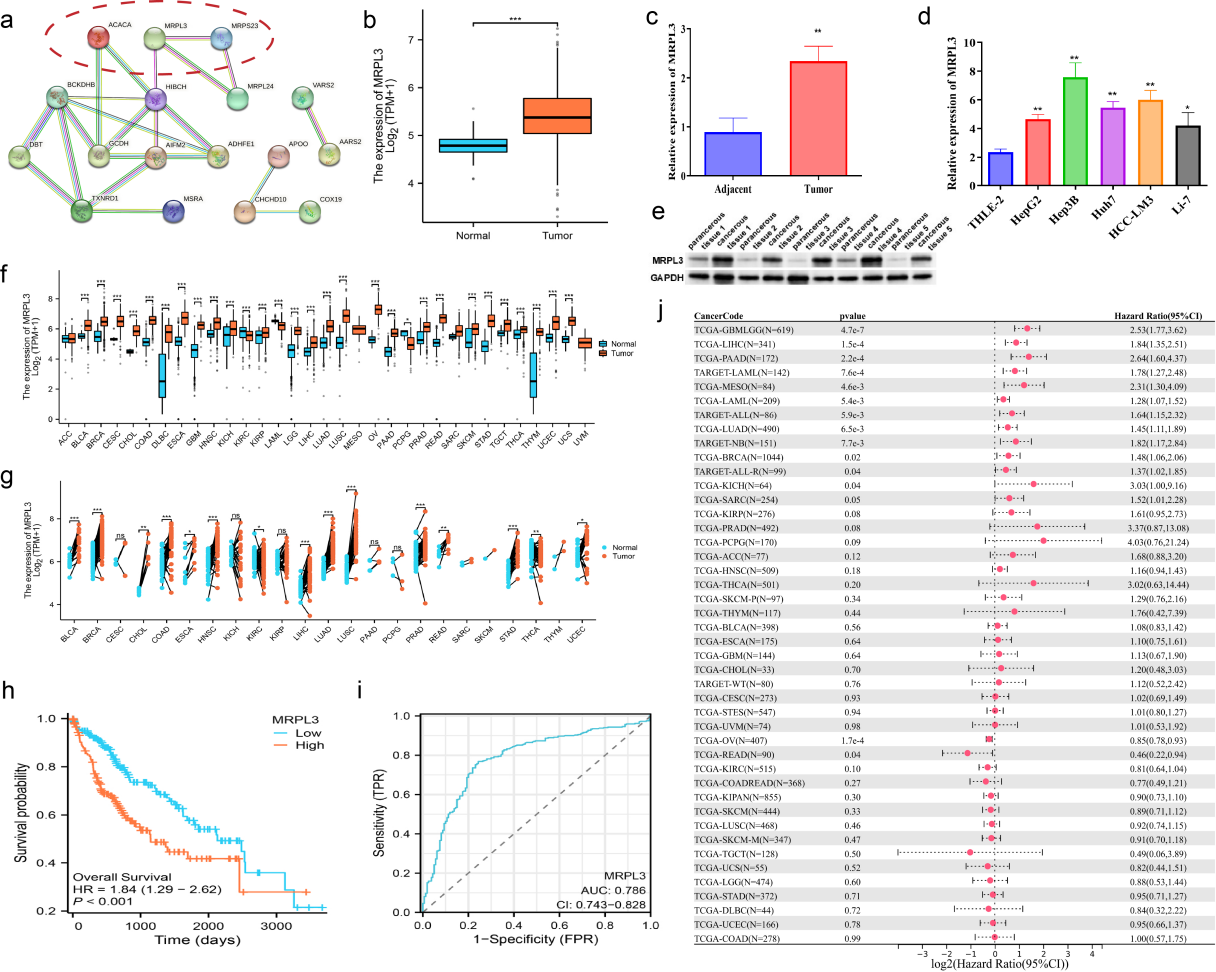
Comprehensive Analysis of MRPL3 Expression, Prognostic Significance, and Diagnostic Value in HCC. **(A)** PPI network of all DE-LMRGs. **(B)** Differential expression of MRPL3 in hepatic normal and HCC tissues according to TCGA dataset. **(C)** RT-qPCR results of MRPL3 in hepatic normal and HCC tissues. **(D)** RT-qPCR results of MRPL3 in hepatic normal cell line and five HCC cell lines. **(E)** Western blot results of MRPL3 in hepatic normal and HCC tissues. **(F)** Expression of MRPL3 in 33 tumors. **(G)** Differential expression of MRPL3 in paired samples. **(H)** Differential expression of MRPL3 in multiple normal and tumor tissues. **(I)** The plausibility of MRPL3 for HCC survival prediction. **(J)** MRPL3 prognostic HR values in multiple tumor types. *p<0.05; **p<0.01; ***p<0.001.

MRPL3 had the highest coefficient value in the LMRG model, and no articles targeting this gene for the treatment of HCC were found. Thus, we performed a single gene analysis for MRPL3. Moreover, we showed the PPI profiles of all DE-LMRGs (**Figure 8A**) and found that HIBCH as a hub protein linked to other proteins that can tightly link the prognosis-related LMRGs (ACACA, MRPL3) to other DE-LMRGs.

Among 33 tumors, we found that MRPL3 showed high expression in most tumors, compared to paracancerous tissue (**Figure 8F**). As paired samples were present in TCGA, we also performed differential expression analysis of MRPL3 in paired samples, and the results were as above (**Figure 8G**). This was also true in TCGA-HCC, with high expression in HCC tissue and low expression in paracancerous tissue (**Figure 8B**). Next, we analyzed the survival profile of MRPL3 in 33 tumors using Hazard Ratio (HR) values to indicate their prognosis and found that MRPL3 was correlated with a poor prognosis in the majority of tumor types (**Figure 8J**), as was the case for TCGA-HCC (**Figure 8H**). MRPL3 demonstrated remarkable predictive efficacy in forecasting the prognosis of HCC patients, with the AUC value was 0.786 (**Figure 8I**). To validate the expression of MRPL3 in HCC, we performed RT-qPCR and Western blot experiments. The results demonstrated a notable elevation in the expression level of MRPL3 in HCC tissues in comparison to normal hepatic tissues (**Figures 8C, E**). Consistent with this finding, cellular experiments revealed that all five HCC cell lines exhibited higher expression of MRPL3 than the hepatic normal cell line (**Figure 8D**). Abbreviations for all tumors are detailed in the supplemental document (**Supplementary Table S3**).

### 3.9 Functional characterization of MRPL3: impact on proliferation, apoptosis, migration, and invasion in HCC cells

**Figure 9:**
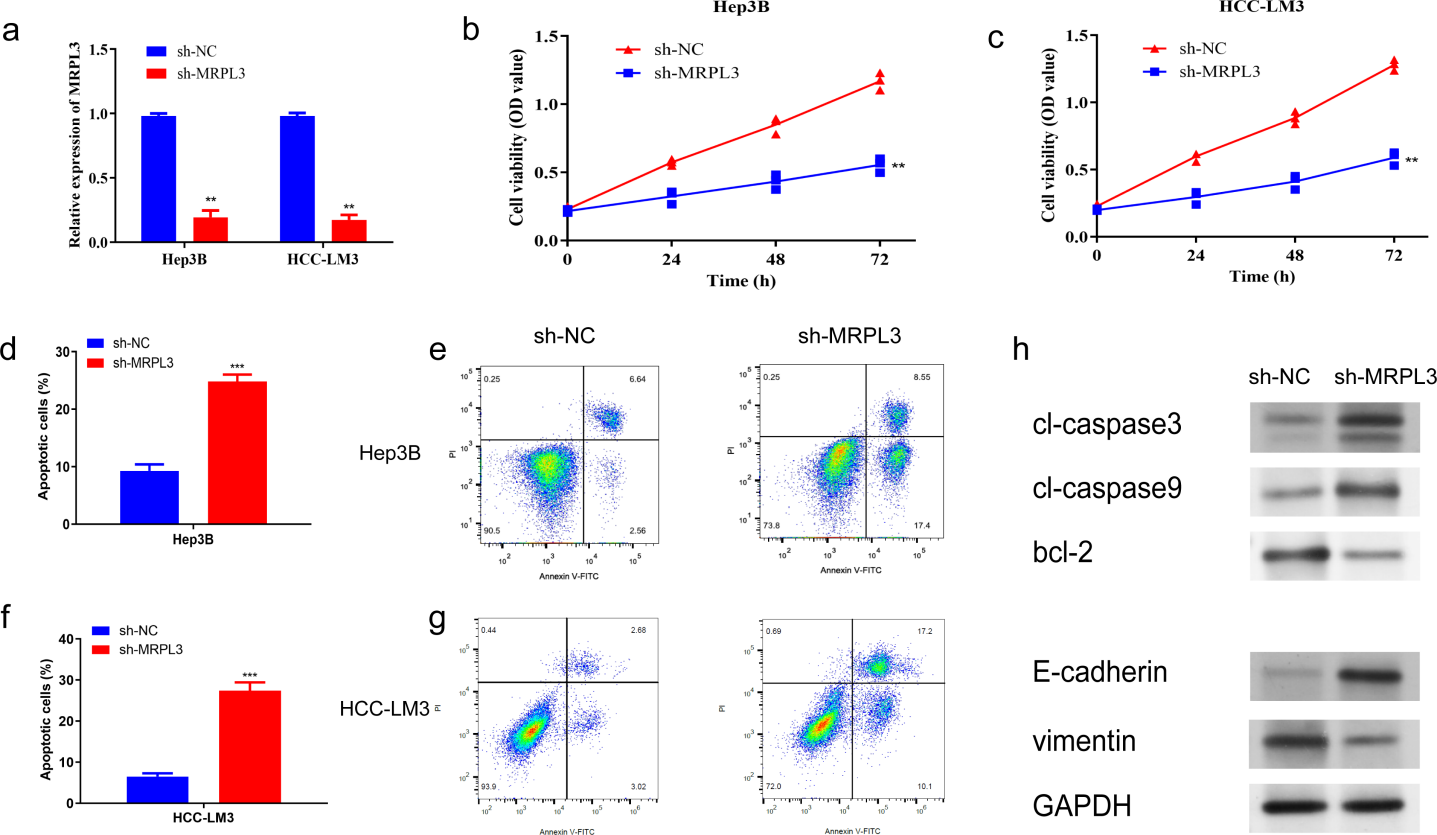
Impact of MRPL3 Knockdown on Proliferation and Apoptosis in Hep3B and HCC-LM3. **(A)** Expression of MRPL3 after silencing. **(B, C)** Silencing of MRPL3 in Hep3B and HCC-LM3 cells, respectively, and CCK-8 assay of cell proliferative activity. **(D, E)** Apoptosis after silencing of MRPL3 in Hep3B cells. **(F, G)** Apoptosis after silencing of MRPL3 in HCC-LM3 cells. **(H)** Western blot detection of MRPL3 apoptosis, migration and invasion related proteins after silencing in Hep3B cells. **p<0.01; ***p<0.001.

To further identify the biological function of MRPL3, sh-RNA was utilized to knockdown MRPL3 in Hep3B and HCC-LM3 cell lines for subsequent experiments (**Figure 9A**). CCK-8 revealed a significant reduction in cell viability following the knockdown of MRPL3 in Hep3B cells, with effective inhibition of cell proliferation observed at 72 hours (p<0.01) (**Figure 9B**). The same result was obtained in HCC-LM3 cells (**Figure 9C**). Flow cytometry also revealed that knockdown of MRPL3 promoted apoptosis in Hep3B and HCC-LM3 cells (**Figure 9D, F**), with a majority of apoptotic cells observed in the late stage (**Figures 9E, G**). Western blotting results (**Figure 9H**) indicated that in Hep3B cells, compared to sh-NC, sh-MRPL3 exhibited significantly increased expression levels of apoptotic proteins (c-caspase3 and c-caspase9). Additionally, there was increased expression of E-cadherin and decreased expression of vimentin, which are associated with tumor suppression and progression, respectively. These findings suggest that knockdown of MRPL3 may not only promote apoptosis in HCC cells but also potentially enhance their invasion and migration.

**Figure 10:**
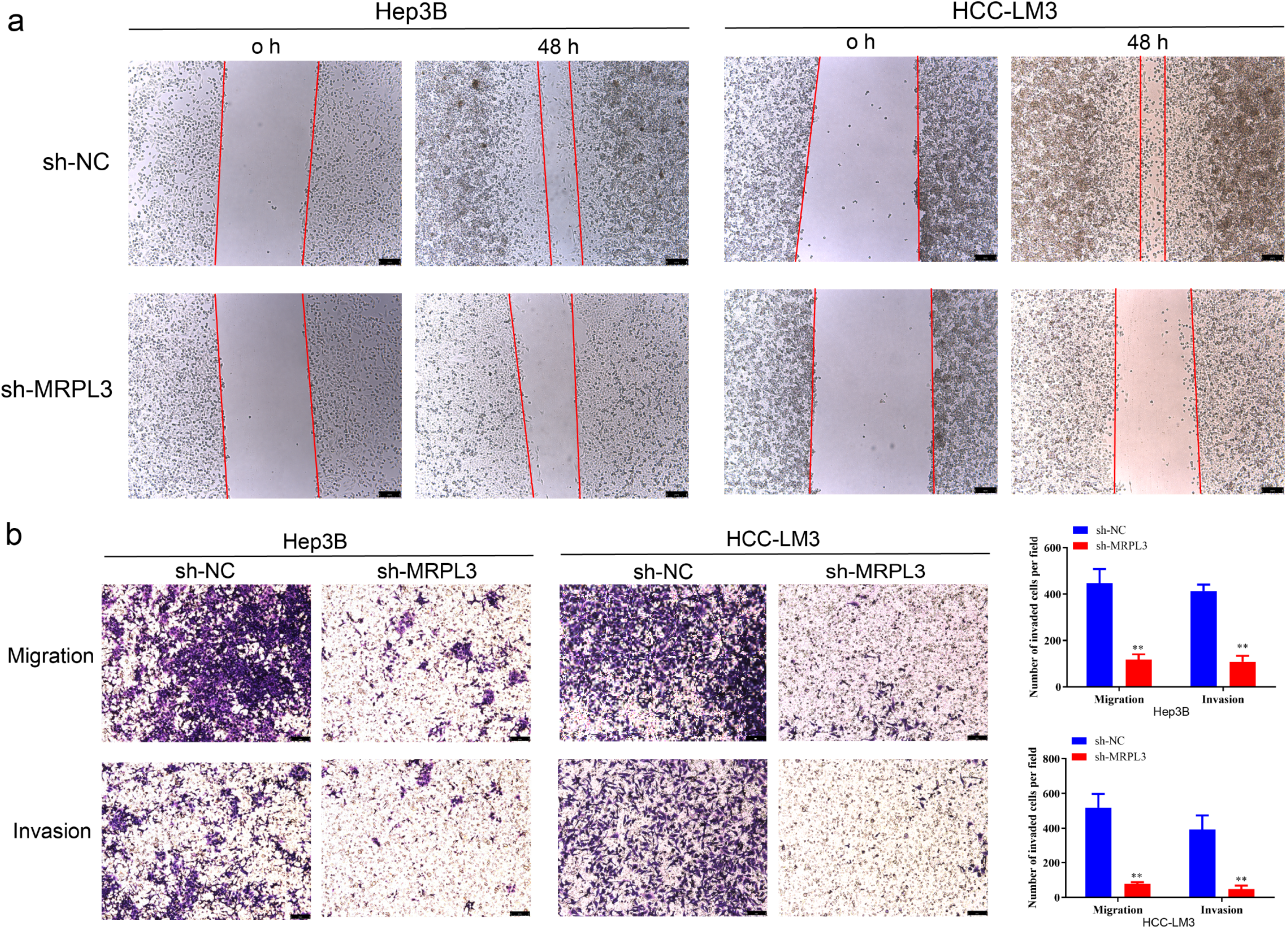
Migration and invasion ability of MRPL3 in HCC cell lines. **(A)** Wound-healing assay of silencing MRPL3 in Hep3B and HCC-LM3 cells. **(B)** Cell migration and invasion ability of silencing MRPL3 in Hep3B and HCC-LM3 cells. **p<0.01.

Furthermore, wound-healing assays revealed that the downregulation of MRPL3 significantly hindered the migratory capacity of Hep3B and HCC-LM3 cells (**Figure 10A**). Transwell assays observed that the knockdown of MRPL3 not only suppressed the migration of the HCC cells but also inhibited the invasion abilities (**Figure 10B**). These experimental results uncover the central biological functions of MRPL3 in HCC.

## 4 Discussion

Lactate, a byproduct of tumor metabolism, plays a dual role in tumorigenesis, progression, and immunosuppression (34). Recent studies have also revealed its epigenetic impact, including histone modification, which regulates gene expression (13). Mitochondria, essential for energy production, are similarly vital for tumor cells. Mutations in mitochondrial genes can drive tumor development, while inhibiting mitochondrial function disrupts tumor metabolism, potentially inducing cell death (35, 36). Under aerobic conditions, pyruvate enters the TCA cycle as acetyl-CoA, but in anaerobic conditions, lactate is produced as an alternative (37). This underscores a strong connection between lactylation and mitochondria. To explore this relationship, we conducted molecular-genetic bioinformatics analyses using public datasets. We identified 82 lactylation-mitochondria-related genes (LMRGs) by intersecting lactylation-related genes from original studies with mitochondria-related genes from MitoCarta3.0.

Lactylation, a recently identified post-translational modification, has been implicated in the metabolic reprogramming of cancer cells. In hepatocellular carcinoma (HCC), lactylation of specific proteins can modulate mitochondrial function, thereby influencing tumor progression. For example, the lactylation of lysine at position K28 of the AK2 protein has been shown to promote HCC deterioration. Additionally, SIRT3-mediated de-lactylation of CCNE2 inhibits liver cancer cell proliferation, underscoring the regulatory role of lactylation in cell cycle control (15). Recent studies have also revealed that histone lactylation is associated with enhanced transcription of mitochondrial biogenesis regulators, linking metabolic reprogramming with epigenetic control (16). These findings suggest that targeting lactylation could offer new therapeutic avenues for HCC treatment (17).

Based on LMRGs, we successfully clustered HCC patients into distinct groups with high intra-cluster consistency. We then developed a prognostic model for HCC using LMRGs, which demonstrated strong predictive accuracy for patient survival. Among the identified prognosis-related LMRGs, ACACA, MRPS23, and MRPL3 emerged as key candidates for HCC diagnosis and treatment. ACACA promotes HCC malignancy by aberrantly activating the Wnt/β-catenin signaling pathway. Its downregulation significantly suppresses HCC cell migration, invasion, proliferation, and EMT, while inducing cell cycle arrest (38). MRPS23 is an independent prognostic marker associated with tumor size, TNM stage, and overall survival (OS). Silencing MRPS23 reduces HCC proliferation both *in vitro* and *in vivo* (39). MRPL3, primarily studied in early embryonic development, impacts ribosome assembly and mitochondrial translation. It has been linked to lymph node metastasis, higher SBR grading, and Ki-67 expression in breast cancer, suggesting a role in tumor proliferation (40–42). However, the connection between MRPL3 and HCC remains largely unexplored, requiring further investigation.

Hence, we conducted a series of analyses for MRPL3, which has the highest coefficient in the LMRG model, to explore its association with HCC. MRPL3, which is fully known as mitochondrial ribosomal protein L3. Whereas mitochondrial ribosomes are found within eukaryotic cells, which are responsible for accomplishing the translation process within an organelle like the mitochondrion (43), its instability and tumor development can lead to a vicious cycle (44). Therefore, MRP family genes can be used as markers for cancer diagnosis and prognostic status (45). We analyzed the expression level of MRPL3 across 33 types of cancer and discovered that it was significantly elevated in tumor tissues for most cancers, including HCC. Furthermore, high levels of MRPL3 expression were associated with poor prognoses in many cancers, such as prostate and colorectal cancer, which was consistent with the bioinformatics analysis of the MRP family by the article of Yu L et al. (46). We probed the correlation between MRPL3 and HCC with *in vitro* experiments. MRPL3 expression was markedly elevated in HCC compared to hepatic normal cells and tissues in RT-qPCR experiments. More promisingly, MRPL3 proved to be a reliable predictor of prognosis in HCC patients. Experimental results demonstrated that MRPL3 knockdown influenced key proteins related to apoptosis, cell proliferation, and migration. A significant downregulation of MRPL3 markedly impeded the growth and migration of HCC cells while simultaneously enhancing their apoptotic response. These researches demonstrated that MRPL3 may serve as a target to inhibit HCC tumor progression, thus providing strong support for clinical decision-making.

To further elucidate the mechanistic relevance of MRPL3 in HCC, we explored its roles in lactylation and mitochondrial functions, which revealed its potential as a crucial mediator in tumor metabolic reprogramming and epigenetic regulation. MRPL3, identified as a key component of the LMRG model, is intricately associated with both lactylation and mitochondrial functions. As a mitochondrial ribosomal protein, MRPL3 is essential for mitochondrial translation and maintaining mitochondrial integrity, a critical factor for oxidative phosphorylation and energy metabolism. Dysregulation of MRPL3 can destabilize mitochondrial ribosome assembly, impairing the electron transport chain and leading to metabolic reprogramming that supports tumorigenesis. Furthermore, MRPL3’s overexpression in HCC tissues and cell lines correlates with metabolic shifts toward glycolysis and lactate accumulation, hallmarks of cancer metabolism. Lactate, in turn, promotes histone lactylation, a process influencing gene expression relevant to tumor proliferation and immune evasion. Our study revealed a significant correlation between MRPL3 expression and poor prognosis in HCC, highlighting its role in metabolic adaptation and tumor progression. PPI analysis further linked MRPL3 with HIBCH, an enzyme vital for mitochondrial amino acid metabolism, suggesting a cooperative role in regulating mitochondrial and lactylation-mediated metabolic pathways. Experimental validation confirmed that silencing MRPL3 disrupted mitochondrial function and inhibited HCC cell proliferation, migration, and invasion. These findings suggest that MRPL3 not only contributes to mitochondrial metabolism but also integrates lactylation-related epigenetic regulation, underpinning its critical role in HCC pathophysiology.

Besides, the PPI network revealed that MRPL3 is not only tightly linked to MRP family genes (MRPS23, MRPL24), but also closely associated with HIBCH (47). Upregulation of HIBCH is shown to be connected with poor prognosis in other tumors (48), which is the same as the upregulation of MRPL3. HIBCH acts as a hub gene associating prognosis-related LMRGs with other DE-LMRGs, and its importance in biological processes cannot be overstated. Research has demonstrated that HIBCH is crucial for amino acid metabolism, with its proper function being closely linked to overall cellular metabolic processes. Mutations in HIBCH may trigger abnormalities in mitochondrial respiratory chain enzymes and pyruvate dehydrogenase, which in turn disrupts respiration and metabolism (49). However, the underlying link between HIBCH and MRP family members is currently under-explored. We venture to speculate in this paper that there may be some as-yet-unknown mechanism of interaction between MRPL3 and HIBCH capable of modulating the progression of tumor and thus interfering with respiration and the TCA cycle.

The LMRG score and MRPL3 both positively correlate with M2 macrophage infiltration, with Chen DY et al. highlighting MRPL3’s role in M2 macrophage polarization (40). M2 tumor-associated macrophages suppress inflammation, promote tumor proliferation, and aid immune evasion (50). Targeting MRPL3 through immunotherapy may help rebalance the M1/M2 ratio by reprogramming M2-like macrophage metabolism, potentially enhancing tumor treatment. The LMRG score also negatively correlates with CD4+ T memory cells, though MRPL3 shows no significant association. This discrepancy may stem from ACACA in the LMRG model, as ACACA deficiency is known to enhance CD4+ T memory cell generation by affecting fatty acid biosynthesis (51). Immune-enhancing drugs might downregulate prognosis-related LMRG expression, reducing HCC incidence. Drug sensitivity analysis identified ML323 as a promising candidate for HCC therapy. As a USP1 inhibitor, ML323 reduces macrophage infiltration, regulates CD4+ T cell differentiation, and inhibits Th17 cell development, maintaining immune balance and exerting anti-tumor effects (52–54). While these findings inform HCC treatment, they require validation through extensive clinical trials. Additionally, our LMRG model, based on public datasets, needs further testing with clinical samples.

Compared to traditional HCC biomarkers such as AFP, DCP, and GPC3, MRPL3 demonstrated superior predictive power and a stronger correlation with advanced clinicopathological features. While AFP is widely used in clinical practice, its sensitivity and specificity are often limited, particularly in early-stage HCC (55, 56). Similarly, DCP and GPC3, although valuable, lack the integrative insights provided by MRPL3 into mitochondrial dysfunction and metabolic reprogramming (57, 58). Our analysis showed that combining MRPL3 with AFP in a composite prognostic model further improved predictive accuracy, emphasizing MRPL3’s additive clinical utility. These results underscore MRPL3’s potential not only as a standalone biomarker but also as a complementary factor in enhancing the prognostic capacity of existing models.

The features of the article are the LMRG-based prognosis-related genes as novel options for the diagnosis and treatment of HCC, with MRPL3 among them available as a new immunotherapeutic target. Patients with HCC can undergo genetic testing based on their LMRG score, allowing for classification in a high- or low-LMRG-score groups so that patients can be given appropriate treatment. This will bring new inspiration for the clinical treatment of HCC.

## Data Availability

The original contributions presented in the study are included in the article/**Supplementary Material**. Further inquiries can be directed to the corresponding authors.
