# Supplementary figures and images for: Multiomic analysis of lactylation and mitochondria-related genes in hepatocellular carcinoma identified MRPL3 as a new prognostic biomarker
Source: Front Oncol. 2025 Jan 10;14:1511958. doi: 10.3389/fonc.2024.1511958 (PMC11757296; doi:10.3389/fonc.2024.1511958)

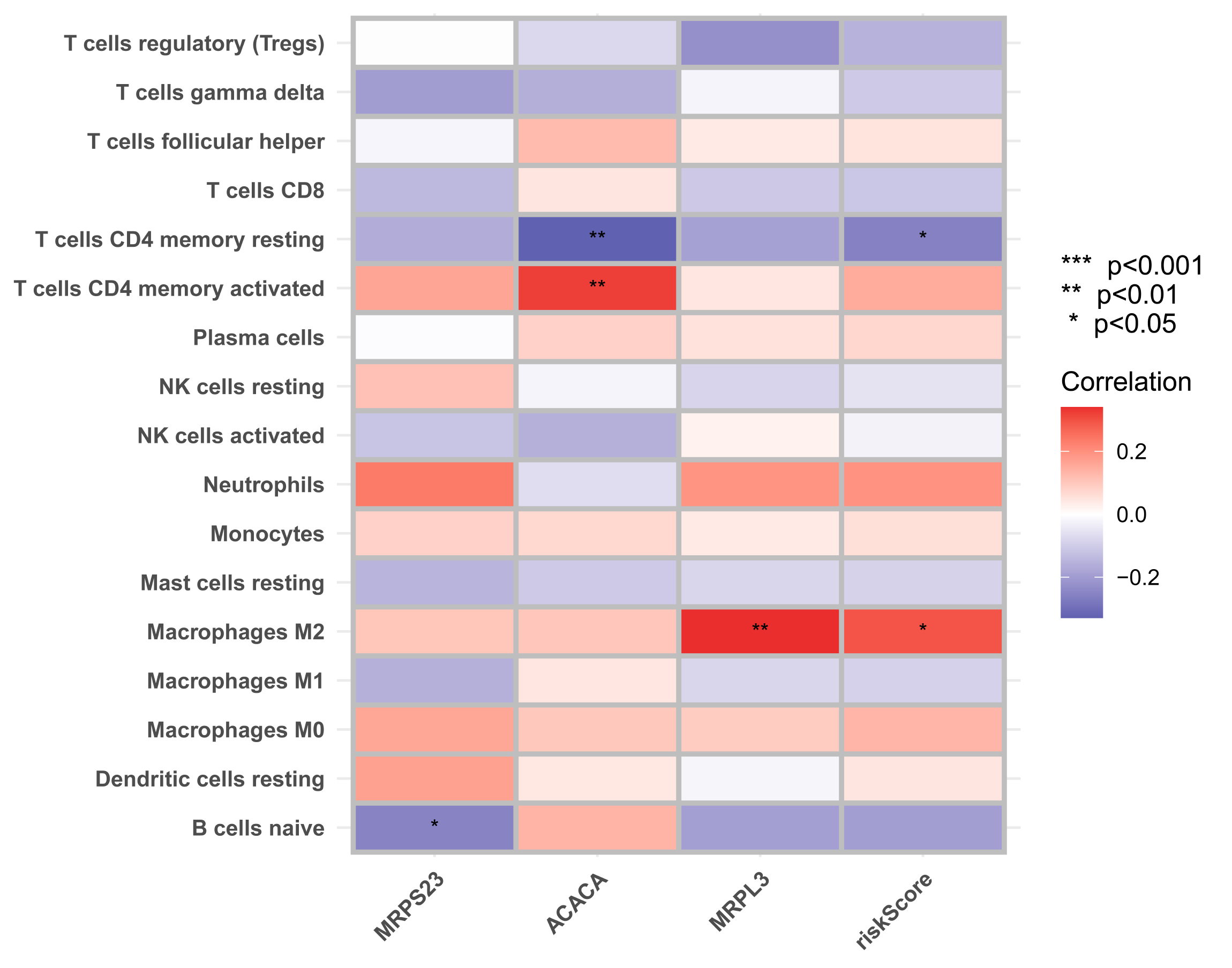

Supplement: Supplementary file 1 [file Image1.tif]

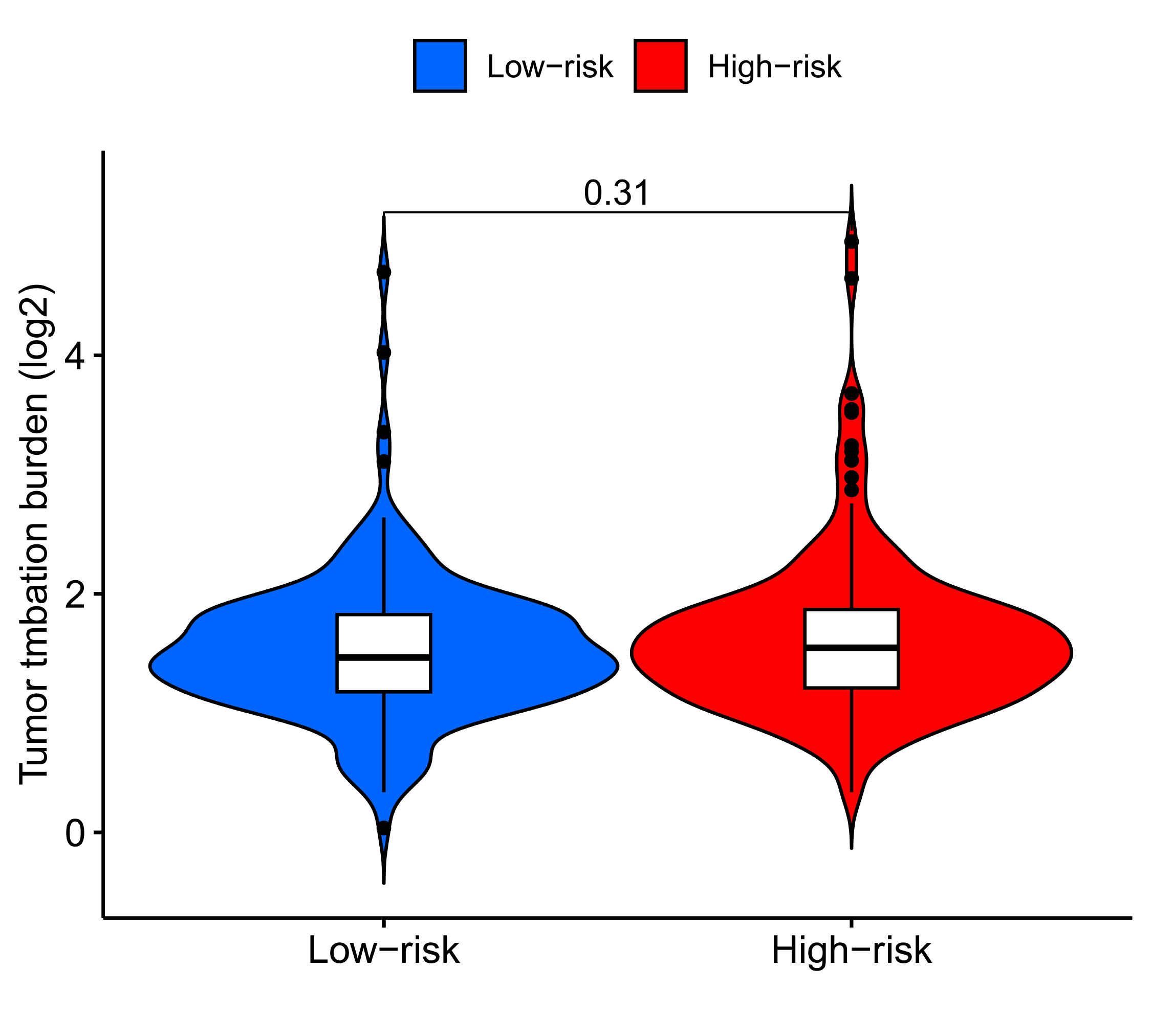

Supplement: Supplementary file 2 [file Image2.tif]

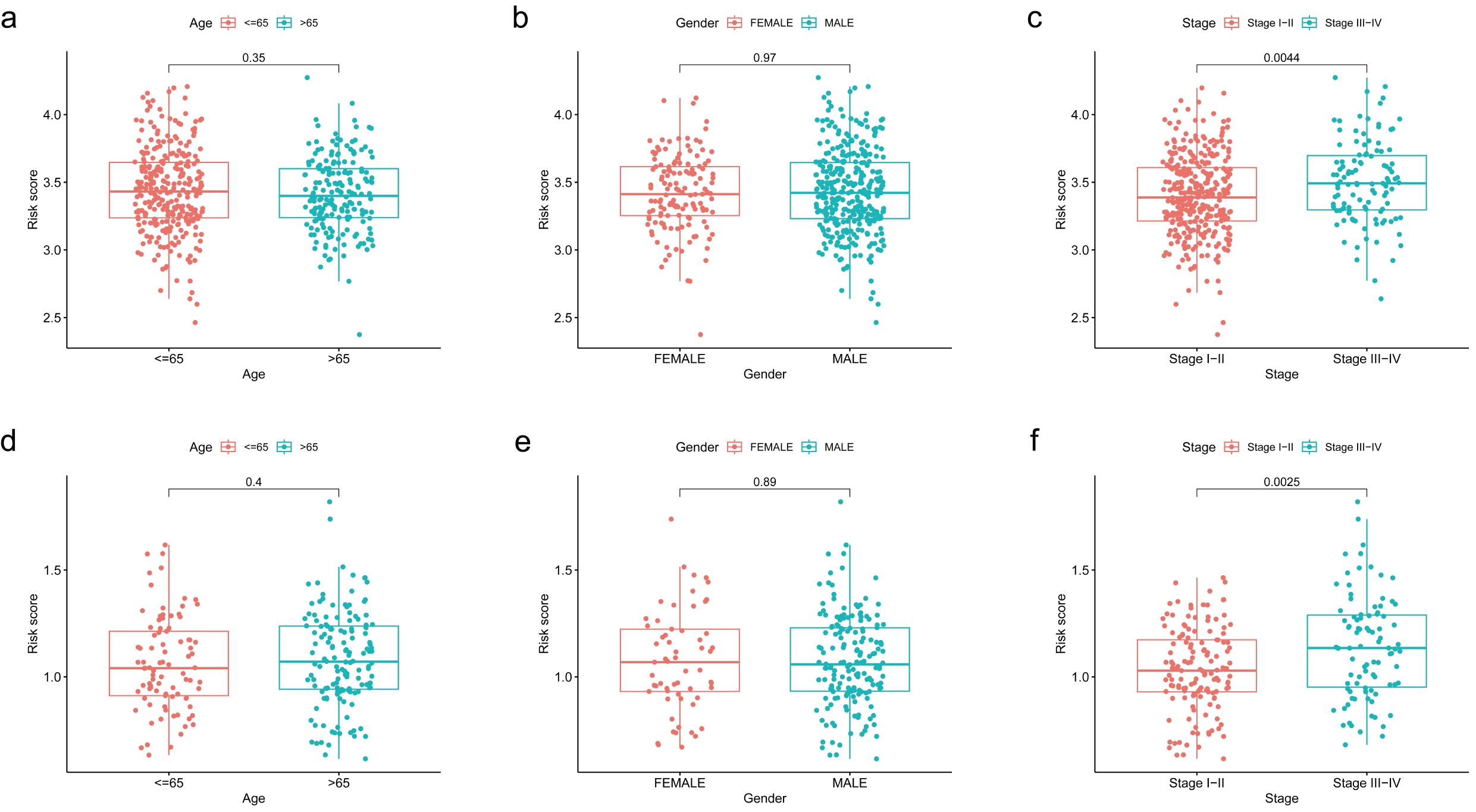

Supplement: Supplementary file 3 [file Image3.tif]
